# Collagen Fibrils Mechanically Contribute to Tissue Contraction in an In Vitro Wound Healing Scenario

**DOI:** 10.1002/advs.201801780

**Published:** 2019-03-14

**Authors:** Erik Brauer, Evi Lippens, Oliver Klein, Grit Nebrich, Sophie Schreivogel, Gabriela Korus, Georg N. Duda, Ansgar Petersen

**Affiliations:** ^1^ Julius Wolff Institute Charité—Universitätsmedizin Berlin 13353 Berlin Germany; ^2^ Berlin‐Brandenburg School for Regenerative Therapies Charité—Universitätsmedizin Berlin 13353 Berlin Germany; ^3^ Berlin‐Brandenburg Center for Regenerative Therapies Charité—Universitätsmedizin Berlin 13353 Berlin Germany; ^4^ Center for Musculo‐Skeletal Surgery Charité—Universitätsmedizin Berlin 13353 Berlin Germany

**Keywords:** cell force, collagen, extracellular matrix, second harmonic imaging, tension, tissue regeneration, traction force microscopy, wound contraction

## Abstract

Wound contraction is an ancient survival mechanism of vertebrates that results from tensile forces supporting wound closure. So far, tissue tension was attributed to cellular forces produced by tissue‐resident (myo‐)fibroblasts alone. However, difficulties in explaining pathological deviations from a successful healing path motivate the exploration of additional modulatory factors. Here, it is shown in a biomaterial‐based in vitro wound healing model that the storage of tensile forces in the extracellular matrix has a significant, so‐far neglected contribution to macroscopic tissue tension. In situ monitoring of tissue forces together with second harmonic imaging reveal that the appearance of collagen fibrils correlates with tissue contraction, indicating a mechanical contribution of tensioned collagen fibrils in the contraction process. As the re‐establishment of tissue tension is key to successful wound healing, the findings are expected to advance the understanding of tissue healing but also underlying principles of misregulation and impaired functionality in scars and tissue contractures.

## 1 Introduction

The contraction of tissue wounds is regarded as a life‐saving mechanism and an essential process during tissue healing, e.g., in skin and many other tissues.^1^, ^2^ The generation of tissue tension or re‐establishment of tissue pretension is believed to be mainly caused by fibroblasts and myo‐fibroblasts, starting already with the traction forces they impose when migrating into the trauma region.^3^, ^4^ As part of the healing process, a fibrous network rich in collagen‐I is established in the injured region that mechanically stabilizes the tissue and shields cells and transitory extracellular matrix components from excessive mechanical stress.^5^, ^6^, ^7^, ^8^, ^9^ So far, tissue contraction and collagen deposition have been regarded as simultaneous, but not as dependent processes in healing. While tissue contraction is attributed to cell traction forces with experimental support from in vitro studies, collagen fibrils are regarded to provide load bearing and stress‐shielding functions subsequent to initial tissue contraction.^10^, ^11^, ^12^ However, first indications for a gradual conversion of cellular tension to the extracellular matrix (ECM) provoke a closer look at a direct involvement of collagen fibrils in tissue tensioning.^3^, ^13^

The complete restoration of tissue functionality after injury is, to a large degree, controlled by the re‐establishment of the ECM. Connected to their functionality, almost all tissues exhibit a basal level of inherent tension often referred to as “resting tension.”^3^ Consequently, to achieve tissue regeneration after injury, the wound is not only required to close, but tissue architecture and intrinsic tension need to be re‐established, usually from a soft and unstructured coagulated blood clot. Fibronectin fibers that play an important role in the early phase of healing were shown to be tensioned by cellular forces with consequences for the deposition of collagen fibrils.^8^ However, the role of tensioned fibronectin fibers in wound contraction and tissue tensioning is unclear. Furthermore, function‐related tension of collagen fibrils in bone was shown to be controlled by the interaction with water.^14^, ^15^ This specific mechanism of developing tension seems to be rather limited to mineralized tissue while detailed insights into the establishment of tension in early stages of bone regeneration and within tissues such as skin, nerves, vessels and ligaments are still missing.

Improper assembly and architectural arrangement of collagen fibrils is associated with pathological conditions. Excessive cellular contraction, e.g., due to chronic myo‐fibroblast activity, is found during hypertrophic scarring in a variety of tissues and organs and is linked to an increased deposition of collagen I.^4^, ^16^ Tissue stiffening compromises cellular functionality which eventually results in tissue or organ failure.^17^ Also, tissue stiffening in primary breast carcinomas was linked to aberrant collagen deposition and structure.^18^ Insights into a potential contribution of fibrillar collagen to tissue contraction and tensioning are lacking despite their value for the identification of strategies for tissue regeneration, but also for preventing tissue fibrosis or malignancy.

In this study, we used a biomaterial‐based in vitro approach together with second‐harmonic imaging to study the role of collagen fibrils in tissue contraction. We found indications for a transfer of cell tensional forces into tensioned collagen fibrils that contributes to tissue tensioning and resulting contraction. Varying the biomaterial stiffness revealed that soft environments (1–3 kPa) could be contracted in absence of collagen fibrils (ascorbic acid depletion), while the contraction of stiffer environments was observed only in the presence of collagen fibrils. Together our data suggests a mechanical contribution of collagen fibrils in macroscopic tissue tensioning during tissue maturation beyond the hematoma phase. These insights might open new routes toward successful tissue regeneration but might also motivate to re‐consider the mechanical role of the extracellular matrix in the development of tissue pathologies.

## 2 Results and Discussion

### 2.1 Synthetic Biomaterial Niche as Model for Wound Healing

In vitro systems used to study and quantify tissue contraction are mostly based on the incorporation of cells into collagen gels.^11^, ^19^, ^20^, ^21^, ^22^, ^23^ Even though deflecting microposts can be used to measure the development of tension in such gels, dissecting the individual contributions of cells and ECM is not trivial due to the gels' complex poroelastic mechanical behavior.^10^ Additionally, available information is limited to short time periods of usually 1–3 d where the deposition of fibrillar collagen cannot be expected.^10^, ^20^ Furthermore, the visualization of fibrillar collagen deposition, e.g., via second harmonic imaging (SHI), is difficult as signals from collagen gel and cell‐secreted collagen fibrils are indistinguishable. Further, hydroxyapatite (HA), polyethylene glycol (PEG) or polydimethylsiloxane‐based (PDMS) biomaterials have been used to study in vitro tissue formation in voids but with limited possibilities to quantify macroscopic tensioning and contraction.^13^, ^24^, ^25^, ^26^, ^27^

**Figure 1:**
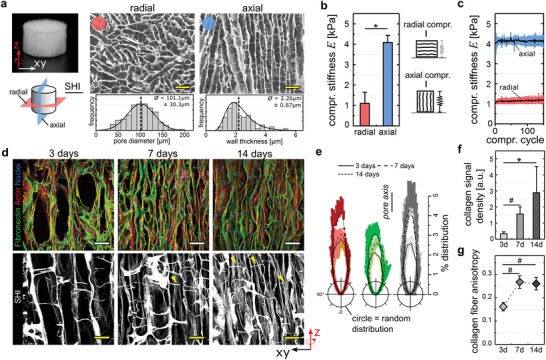
A structured collagen scaffold directs cell organization and ECM formation. a) Visualization of the highly organized cylindrical shaped scaffold (5 mm Ø) (top left). Pictographic representation indicating radial (red) and axial (blue) direction of view (bottom left). Center and right panels show SEM images of pore architecture in radial (red dot) and axial direction (blue dot) including structural analysis of pore diameter (lower left) and wall thickness (lower right). Scale bar 250 µm. b) Compressive stiffness *E* of collagen scaffolds measured perpendicular (radial) or in the direction (axial) of the pores (mean ± S.D., *n* = 4). c) Compressive stiffness plotted over compression cycles (*n* = 4). Black lines indicate the mean with blue/red belt as standard deviation. d) Confocal images of collagen scaffolds imaged in axial direction after 3, 7, or 14 d of culture. Samples were stained for fibronectin (green), actin (red), or cell nuclei (blue). Fibrillar collagen was visualized by SHI (lower panel). Yellow arrows indicate little influence of struts on structural alignment of collagen fibrils at later stages of culture. Scale bar 100 µm. e) Circular plots indicating the orientation distribution of actin (red), fibronectin (green), and collagen fibrils (grey) after 3,7, or 14 d of culture relative to the local pore orientation. The means are reflected as dark lines with standard deviation as colored belt (*n* = 2–4). f) Quantification of collagen signal density (a.u., arbitrary units) inside scaffold pores (mean ± S.D., *n* = 4–9). g) Quantification of the local anisotropy of fibrillar collagen signal inside scaffold pores (mean ± S.D., *n* = 4). Significance levels were calculated using the Mann–Whitney U test (two‐sided) with the Bonferroni correction for comparison of multiple groups. Significance levels indicate # *p* < 0.1, * *p* < 0.05.

In this study we used macroporous scaffolds fabricated from porcine collagen by a directional freezing and freeze‐drying process (1.5 wt/wt% collagen content, Matricel GmbH, Herzogenrath, Germany) as cell carriers.^28^ The scaffolds featured an anisotropic architecture with channel‐like pores along the cylinder axis (axial direction) and an isotropic pore pattern perpendicular to the direction of the channels (radial direction) (**Figure** **1**a). We quantified a mean wall spanning distance of *D* = 101 ± 30 µm (mean ± S.D.) and a wall thickness of *T* = 2.3 ± 0.9 µm. Mono‐axial compression testing was used to characterize the scaffold's mechanical stiffness revealing a significant difference between axial compression along (*E*_axial_ = 4.1 ± 0.4 kPa) and radial compression perpendicular (*E*_radial_ = 1.1 ± 0.5 kPa) to the pore orientation (Figure 1b). Most importantly, the material featured a spring‐like, elastic deformation behavior with full restoration of the initial dimensions and stiffness after multiple repetitive loading cycles (Figure 1c and Figure S1a,b, Supporting Information). Together, the soft, elastic and open‐porous environment and the good spatial discrimination between the biomaterial and cell‐secreted ECM was essential for the in situ measurement of tissue tension and to study the mechanical role of the ECM in this process.

Cylindrical scaffolds (5 mm Ø, 3 mm height) were seeded with primary human dermal fibroblasts (hdFs), as they represent the most relevant cell type to study wound contraction and tissue formation. The dip‐in seeding procedure led to a homogeneous cell distribution and a uniform tissue formation process throughout the scaffold (Figure S1c,d, Supporting Information). An increasing alignment of hdFs and early deposited fibronectin fibers along the direction of the scaffold pores was found at 3, 7, and 14 d of culture (Figure 1d,e). Wall‐connecting struts that are part of the scaffold architecture were used by cells for the initial centripetal pore filling (day 3) but were of diminishing importance for cell and ECM organization when a dense and highly aligned cell network formed within the scaffold pores (7 and 14 d).

Aside of depositing fibronectin and other early ECM components, fibroblasts express and secrete significant amounts of collagen.^29^ As particularly fibrillar collagen exhibits a mechanical load bearing and stress shielding function with potential relevance for tissue tensioning, we visualized collagen fibrils by second harmonic imaging (SHI) (Figure 1d, lower panel). Only individual collagen fibrils were identified after 3 d of culture but over time, a dense network of fibrillar collagen formed that followed the structural alignment of cells and fibronectin fibers along the scaffold pores (Figure 1e–g). Collagen fibrils were spanning over long distances throughout the sample, again with little structural distortion by wall‐connecting struts (Figure 1d, yellow arrows). The limited contact to the scaffold material and the long‐range organization indicated that the tissue formed inside the scaffold pores collectively organized on a macroscopic scale. Such a long‐range organization of cells and ECM was observed previously in vitro and in vivo.^11^, ^30^

Taken together, we observed a time‐dependent cellular self‐organization and a consecutive deposition of fibronectin and fibrillar collagen within the channel‐like scaffold pores resulting in a dense, highly aligned ECM network with almost identical structural properties as the cell‐network. The unidirectional character of the cell‐ECM network was advantageous for the subsequent analysis of tissue tension as the mechanical interplay of cell/ECM tension acting against scaffold wall compression was mostly reduced to one spatial dimension—the direction of the scaffold pores. The uniform thickness of the parallel scaffold walls and their elastic deformation provided a spring‐like substrate with very homogeneous stiffness in the direction of the pores (=axial direction). This was a prerequisite for the subsequent calculation of tissue tensional force based on scaffold strain.

### 2.2 In Vitro Tissue Contraction Depends on the Presence of Collagen Fibrils

**Figure 2:**
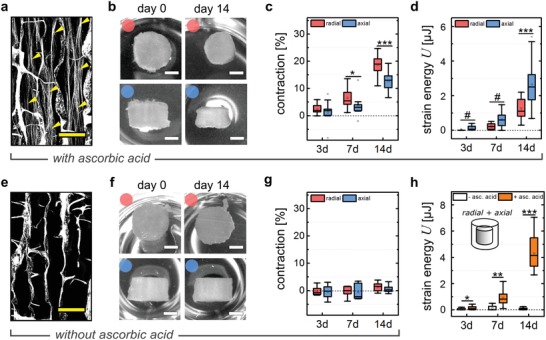
Macroscopic contraction depends on the deposition of a fibrillar collagen ECM. a) SHI of microtissues after 14 d of culture including ascorbic acid supplementation. Yellow arrowheads indicate cell‐produced collagen fibrils. Scale bar 100 µm. b) Representative scans of seeded scaffolds for axial (blue dots) and radial (red dots) directions after seeding (day 0) and day 14 of culture with ascorbic acid supplementation. Scale bar 2 mm. c) Quantification of scaffold contraction in axial and radial directions expressed as percent of initial volume (day 0) for 3, 7, and 14 d of culture with ascorbic acid supplementation (*n* = 13–24). d) Calculated strain energy levels of the collagen scaffold as a result of macroscopic contraction both for radial (red) and axial (blue) direction (*n* = 7–19). e) SHI of microtissues after 14 d of culture without ascorbic acid supplementation. Scale bar 100 µm. f) Representative scans of seeded scaffolds for axial (red dots) and radial (blue dots) directions after seeding (day 0) and day 14 of culture without ascorbic acid supplementation. Scale bar 2 mm. g) Quantification of scaffold contraction in axial and radial directions expressed as percent of initial volume (day 0) for 3, 7, and 14 d of culture without ascorbic acid supplementation (*n* = 8). h) Calculated strain energy levels (total sample) as a result of macroscopic contraction cultured either in the presence or absence of ascorbic acid (*n* = 8–14). Significance levels via the Mann–Whitney U test (two‐sided) and significance levels are indicated by symbols: * *p* < 0.05, ** *p* < 0.01, *** *p* < 0.001.

It was reported before that cell traction forces are able to induce a macroscopic deformation of biomaterial scaffolds in vitro.^11^, ^19^, ^22^, ^31^, ^32^ In agreement with this, we observed a time‐dependent scaffold contraction, both in axial and radial direction, under supplementation of ascorbic acid that is required for collagen fibrillogenesis (**Figure** **2**a). Scaffold contraction was still weak at day 3 but showed a pronounced increase until day 14. At the same time radial contraction was significantly higher compared to axial contraction (Figure 2b,c). As this observation was contradictory to the high cell and ECM alignment along the scaffold pores we assessed the biomaterial deformation in a mechanically quantitative manner by calculating scaffold strain energies taking into account the higher compressive stiffness of the scaffold in axial compared to the radial direction. Consequently, and in agreement with cell and ECM orientation, we found significantly higher strain energies in the axial compared to the radial direction after 14 d of culture (Figure 2d). To exclude potential changes of the biomaterial's macroscopic stiffness due to creep, we gradually compressed empty scaffolds using a mechano‐bioreactor system described before mimicking the situation during culture of cell‐seeded scaffolds.^33^ No alterations of scaffold stiffness were found when reaching 13% compressive strain representing the mean strain in axial direction exerted by cells over 14 d (Figure S2a, Supporting Information).

Assuming that cells initiate contraction of the environment by the application of traction forces alone, scaffold contraction would occur even in the absence of collagen fibrils. To test this, the formation of fibrillar collagen inside scaffold pores was suppressed by the depletion of ascorbic acid from the culture medium. While cell adhesion, proliferation and deposition of fibronectin fibers remained unaffected, a significant reduction in collagen density and pore filling was observed (Figure 2e, Figure S2b–d, Supporting Information). Most strikingly, samples cultured in the absence of ascorbic acid completely lacked macroscopic scaffold contraction over the 14 d of culture (Figure 2f,g). This was also reflected by significantly reduced values of total scaffold strain energy (radial + axial contributions) in the ascorbic acid‐depleted groups for all analyzed time points (Figure 2h).

Together these findings provide evidence that collagen fibrils are essential elements in pore filling and tissue tensioning. The fact that measurable scaffold compression (straining) is taking place only when collagen fibrils are present was a first hint toward their central role for creating intrinsic tissue tension.

### 2.3 Indications for a Transfer of Cell Tensional Forces into the Fibrillar Collagen Network

Assuming that cellular forces can be added up linearly due to the high cellular alignment along the pores, the maximal force would be the sum of all parallel‐acting individual cell forces. Consequently, macroscopic contraction would correlate with the contractile capacity of single cells.

**Figure 3:**
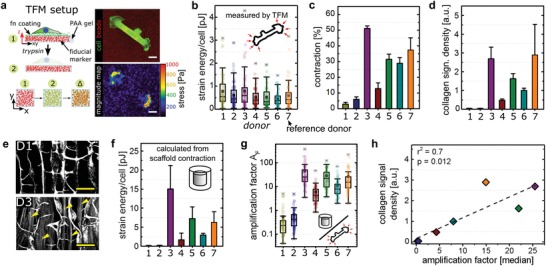
Fibrillar collagen amplifies single cell traction forces. a) Pictographic representation of TFM procedure based on the displacement of fiducial markers. Right: original image with fluorescent beads of 100 nm size (red) and cell (green) and calculated force magnitude map. Scale bar 20 µm. b) Single cell strain energies of at least 60 cells from three independent experiments measured by TFM for seven primary donors. Each colored dot represents the calculated value of a single cells. c) Quantification of scaffold contraction (total volume) of seven primary donors cultured with ascorbic acid after two weeks of culture (mean ± S.D., *n* = 3–9). d) Quantification of fibrillar collagen signal density of scaffolds seeded with seven different primary donors after two weeks of culture (mean ± S.D., *n* = 3–7). e) SHI of scaffolds seeded with hdFs derived from donors 1 or 3 and cultivated for 14 d in the presence of ascorbic acid. Yellow arrowheads indicate cell‐secreted collagen fibrils. Scale bar 100 µm. f) Calculated strain energy levels of total samples as a result of macroscopic contraction which were normalized to the total cell number. This results in values of strain energy per cell related to macroscopic contraction (mean ± S.D., *n* = 3–9). g) Calculated amplification factor for at least 60 cells reflecting the ratio between macroscopic strain energy per cell and the single cell force. h) Correlation of fibrillar collagen density and the median of calculated amplification factors indicating a positive linear correlation (*r* ^2^ = 0.7).

Speculating that primary fibroblasts from different donors vary in their ability to generate traction forces, we expected additional insights into the dependency between cell tension and tissue tension from an interdonor comparison. We isolated primary dermal fibroblasts from seven different human donors (age 24.9 ± 6.9 years, mean ± S.D.) and performed single cell force measurements by traction force microscopy (TFM) (**Figure** **3**a).^34^, ^35^ Surprisingly, aside of a strong intrinsic variation for each individual donor, we detected only small differences between donors with mean values ranging from 0.44 (donor D6) to 0.76 pJ per cell (donor D1) (Figure 3b, Figure S3a, Supporting Information).

We next cultured fibroblasts from all seven donors individually inside collagen scaffolds and quantified scaffold contraction after 14 d of culture (Figure 3c). Remarkably, we observed a pronounced, up to 17‐fold variation in the magnitude of scaffold contraction which did not correlate with differences in single cell forces (Figure S3b, compare donors D1 and D3, Supporting Information). As the depletion of ascorbic acid and the resulting lack of collagen fibrillogenesis had shown to abolish scaffold contraction, we hypothesized that a variation in the amount of collagen fibrils might be responsible for the observed differences in contraction. Indeed, fibrillar collagen density inside the scaffold pores showed an almost identical variation between the donors as it was observed for scaffold contraction (Figure 3d). Correlating collagen density and scaffold contraction revealed a direct linear dependency (Figure S3c in the Supporting Information, *r* ^2^ = 0.85).

In order to estimate the mechanical contribution of an individual cell to the macroscopic scaffold contraction, we calculated scaffold strain energy based on the biomaterial mechanical properties and normalized this to the total number of cells (Figure 3f). While the obtained values do not reflect actual in situ 3D single cell forces (neglecting compression of cell‐secreted ECM components) or 2D cell forces quantified by TFM (not mimicking the physiological cell morphology and environment), they allow an estimation of the contribution of each cell to the macroscopic contraction. We further calculated a ratio of macroscopic scaffold strain energy and microscopic single cell strain energy (based on TFM data) which we termed force amplification factor *A*_F_ (Figure 3g). Intriguingly, we observed that the two donors that exhibited the lowest mean values (*A*_F_ = 0.5 for D1 and 0.6 for D2) of force amplification also showed a complete lack of fibrillar collagen (Figure 3d,e). All other donors with visible collagen fibrils showed mean values ranging between *A*_F_ = 6 (D4) and *A*_F_ = 46 (D3). This indicated that fibrillar collagen strongly contributes to the generation of intra‐tissue tension. The linear correlation between the amplification factor *A*_F_ and collagen fibril density further suggested that cells incrementally transfer tensional forces into the fibrillar collagen network (Figure 3h). In order to exclude a potential derogation of the results through a potential divergence of single cell forces during 3D culture, we characterized fibroblasts activated by 10 ng mL^−1^ TGF‐β1 (Figure S3d,e, Supporting Information). We observed that resulting αSMA‐positive fibroblasts featured a rather mild, approximately twofold increase of traction force compared to unstimulated controls in agreement with previously published data.^36^ Finally, supplementation with ascorbic acid did not alter cell traction forces excluding systematic deviations between the groups investigated.

Taken together, the data indicated that the ability to generate tissue tension and contraction not necessarily depends on the ability to generate high cellular traction forces but rather on the capability to transfer the generated forces into a network of pretensioned collagen fibrils.

### 2.4 Relevance of Collagen Fibril‐Associated Tension during Wound Healing

**Figure 4:**
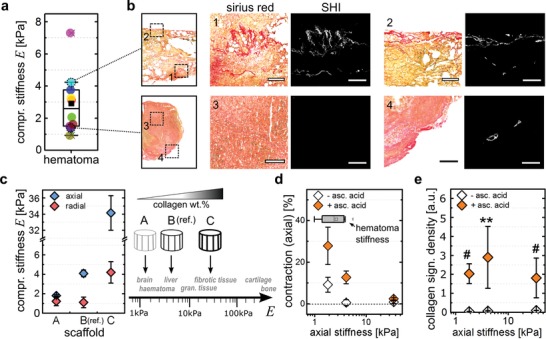
Scaffold stiffness determines cellular strain output. a) Compressive stiffness of human fracture hematoma measured by monoaxial compression testing. Each colored dot represents the stiffness of 1 donor sample (10 in total). b) Histological sections of human fracture hematoma analyzed by Sirius red staining and SHI for visualization of collagen fibrils. Scale bar 200 µm in close‐up views (1–4). c) Compressive stiffness of three different scaffolds (mean ± S.D., *n* = 3 each) produced from a collagen suspension of varying solid content (1.1–3.0 wt%). The compressive stiffness was measured both in axial (compression along the pores, blue) and radial (compression perpendicular to the pores, red) directions of the structured scaffolds. d) Quantification of scaffold axial contraction for hdFs cultured over 2 weeks inside the three prototypes (mean ± S.D., *n* = 3–9) either with or without the supplementation of ascorbic acid. Contraction is plotted over compressive stiffness (axial) of the scaffold prototypes A, B, and C. e) Quantification of fibrillar collagen density after culture of hdFs inside scaffold prototypes (mean ± S.D., *n* = 3–9) for 2 weeks either with or without the supplementation of ascorbic acid. Fibrillar collagen density is plotted over axial stiffness of the scaffold prototypes A, B, and C. Statistics via the Mann–Whitney U test (two‐sided). Significance levels are indicated as: # *p* < 0.1., ** *p* < 0.01.

In an injury situation, the repair‐process including tissue contraction usually starts from a blood clot that forms directly after trauma, e.g., bone fracture. To gain insights into the mechanical properties of this early healing environment, we performed mechanical compression tests on ten human bone fracture hematoma harvested either from proximal humerus or closed acetabulum fractures. We found a large inter‐donor variability with a compressive stiffness ranging from *E* = 0.9 to 7.2 kPa and a mean of *E*_mean_ = 2.9 ± 1.9 kPa (±S.D.) (**Figure** **4**a). Visualization of collagen fibrils via histological Sirius Red staining and SHI revealed a cell‐ and ECM‐rich tissue environment but only sporadically occurring collagen fibrils (Figure 4b).

To gain insights into the relevance of collagen fibrils for tissue contraction at different stages of healing, we aimed to study contraction in different mechanical environments featuring the mechanical stiffness of the hematoma (<2 kPa), of intact soft tissues such as kidney or liver (4–5 kPa) and of maturing granulation tissue (25–50 kPa).^4^, ^37^ By adjusting the collagen solid content of the scaffolds, an additional soft scaffold (scaffold A, 1.1 wt%, *E*_axial_ = 1.7 kPa) and an additional stiff scaffold (scaffold C, 3.0 wt%, *E*_axial_ = 34 kPa) were produced next to the existing material (scaffold B, 1.5 wt%, *E*_axial_ = 4.1 kPa) (Figure 4c). Scanning electron microscopy (SEM) revealed a similar pore architecture of the different prototypes featuring aligned channel‐like pores. Pore diameters were comparable between groups while the increase of wall thickness with increasing solid content was responsible for the increase in scaffold stiffness (Figure S4a–c, Supporting Information). Although a higher density of wall‐connecting struts was visible in the stiffer scaffold C, cell and collagen fiber alignment along the pores was comparable (Figure S4d, Supporting Information).

We analyzed scaffold contraction after 14 d of culture, both in the presence or absence of fibrillar collagen controlled by ascorbic acid supplementation. As differences in stiffness of the scaffold prototypes were higher in axial compared to radial direction, and as cells were predominantly orientated along the pores, the analysis of scaffold contraction was focused on the axial direction. As expected, the ability of the cells to contract the scaffold decreased with increasing scaffold stiffness (Figure 4d). In the absence of collagen fibrils, cells were only able to contract scaffold A mimicking the soft hematoma environment. Remarkably, in the presence of collagen fibrils not only scaffold A, but also scaffold B representing intact soft tissues was significantly contracted. In stiff scaffolds C representing matured granulation tissue only a minor contraction was observed even in the presence of collagen fibrils. This suggested that only through the transfer of tension into the collagen fiber network, sufficient tension can be build up to mechanically contribute to tissue maturation from the hematoma phase toward the restoration of the intact tissue's mechanical properties. Tensional forces applied by the cells alone or with contributions from matrix components other than collagen‐I (e.g., fibronectin) seem to be limited to very early stages of healing where the stiffness of the environment is low (*E* < 4 kPa). However, even in the presence of fibrillar collagen, tension‐controlled contraction seems to reach a limit at a stiffness between 10 and 30 kPa in vitro. Interestingly, SHI showed that scaffold stiffness did not influence collagen fibril density (Figure 4e, Figure S4f, Supporting Information). This might indicate that the limited contraction of stiff scaffolds could be a consequence of a limited collagen fibril density under in vitro conditions. Here, the supply with nutrients and oxygen inside the scaffold is limited at long culture times—in contrast to a well‐vascularized tissue in vivo.

Together, the use of scaffolds mimicking the mechanical environment at different stages of soft tissue healing revealed a fundamental role of collagen fibrils in the mechanical maturation of tissues beyond the hematoma phase.

### 2.5 Collagen Fibrils Carry Mechanical Tension Independent of Active Cell Forces

**Figure 5:**
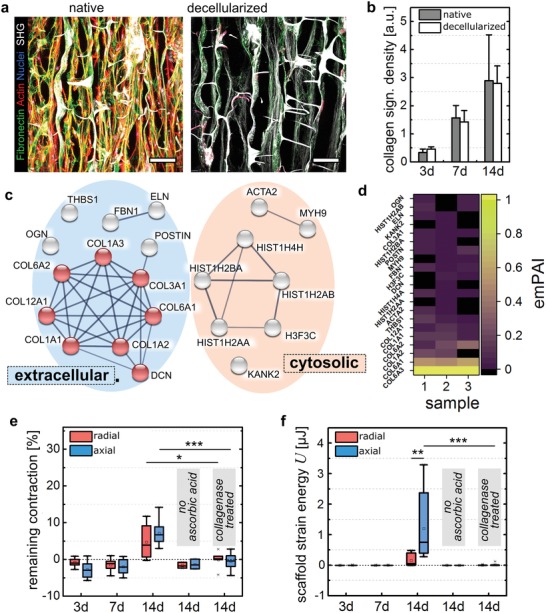
Fibrillar collagen stores cell‐generated traction forces. a) Confocal images of hdFs seeded into scaffolds and cultured for 14 d either before (native) or after (decellularized) decellularization. Samples were stained for fibronectin (green), actin (red), and nuclei (blue). Scale bar 100 µm. b) Quantification of fibrillar collagen density inside scaffold pores after 3,7, or 14 d of culture before and after decellularization (mean ± S.D., *n* = 4–9). c) Functional enrichment analysis of detected proteins that were identified by mass spectrometry (GO:0005581, collagen trimer, red spheres). Lines illustrate interactions based on experiments, databases, coexpression, and co‐occurrence in which the thickness depends on strength of data support. Minimum required interaction score: 0.7. d) EmPAI (exponentially modified protein abundance index) presented as heat map for three independent samples. e) Quantification of contraction after decellularization both for radial (red) and axial (blue) view (mean ± S.D., *n* = 3–18). f) Calculated strain energies derived from scaffold contraction both for radial and axial view (mean ± S.D., *n* = 3–18). Statistics via the Mann–Whitney U test (two‐sided). Significance levels are indicated as: * *p* < 0.05., ** *p* < 0.01, ***p < 0.001.

Our data indicated that tissue contraction results from cell tensional forces being incrementally transferred into a tensioned collagen fibril network. Consequently, tissue stability and tensional state would be independent of active cell forces. In order to test if cell‐secreted fibrillar collagen exhibited a load bearing capacity, we removed cellular components from the 3D scaffold by detergent‐based decellularization. The depletion was validated by histology and confocal microscopy (Actin, Nuclei) as well as residual DNA quantification after decellularization (**Figure** **5**a, Figure S5a, Supporting Information). Immunohistology showed only a faint signal remaining for fibronectin while second harmonic generation (SHG) signal quantification demonstrated no negative effect of the decellularization procedure on collagen density and only minor effects on collagen fiber alignment (Figure 5b, Figure S5b–d, Supporting Information). We further validated the protein composition of the decellularized extracellular matrix (dECM) by liquid chromatography/electron spray ionization mass spectrometry (LC/ESI–MS).^38^ In total 21 different proteins were detected (Tables S1 and S2, Supporting Information) and collagens including type I, III, VI, and XII were identified resembling almost 80% of all detected peptides (Figure 5c,d). Residual cellular components such as histone and acto‐myosin are regarded as debris from the decellularization process which was further confirmed by western blotting to demonstrate the efficient removal of actin and histone cellular components (Figure S5e, Supporting Information). Altogether, these data indicated that collagenous ECM was the main component retained after decellularization.

After 3 and 7 d of culture, no remaining contraction of the scaffold was visible upon decellularization indicating that collagen fibrils did not prevent the relaxation of the scaffold to its original shape (Figure 5e). Intriguingly, after 14 d of culture we detected a remaining contraction after decellularization relative to contraction observed with native samples both for radial (19%) and axial (57% remaining contraction) direction. When taking into account the different stiffness of the scaffold in axial and radial direction, a significantly higher strain energy was found to be stored in the axial direction of the scaffold (Figure 5f). As tensioned collagen fibrils seem to be necessary to hold the scaffold in a compressed state, the directionality of strain energy storage could be explained by the high alignment of collagen fibrils in the axial direction (Figure S5c–d, Supporting Information). Interestingly, the alignment of the collagen fibrils that results from the specific architecture of the scaffold pores is increased after decellularization (Figure S5c, day 3 and day 14, Supporting Information). This indicates that the collagen fibril network is straightened with removal of the cells, potentially as a consequence of eliminating radial components of cell tensional forces or because cell‐occupied inter‐fibril space was made available by decellularization. Fibril network straightening and resulting lengthening upon decellularization might also explain the lack of remaining contraction after shorter time of culture (especially day 7) where scaffold strain in the axial direction was still low (mean contraction 3.5%, Figure 2c). Mechanical characterization of scaffolds after decellularization verified that the scaffold stiffness was not altered over the time period of culture (Figure S5f, Supporting Information). This is of relevance for the interpretation of the scaffold contraction data but also for the calculation of the strain energy stored inside the collagen fibril network. To further demonstrate that the contraction remaining after decellularization was truly a result of collagen fibril tension rather than potential plastic deformation of the scaffold material, we treated decellularized scaffolds with collagenase in a concentration that completely degraded any cell‐secreted ECM, but not the scaffold itself (Figure S5g, Supporting Information). Doing so, we observed a complete removal of remaining contraction and the release of the stored scaffold strain energy (Figure 5e,f). This demonstrates that the cell‐secreted collagen fibril network in our in vitro model exhibited a load‐bearing capacity that would be able to carry cell‐traction forces upon transferred into the fibril network.

### 2.6 Collagen Fibrils—an Underestimated Regulator of Tissue Tension and Contraction?

**Figure 6:**
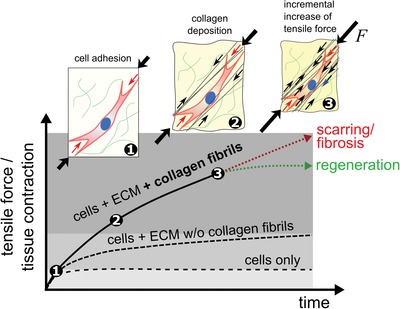
Fibrillar collagen amplifies single cell forces. Graphical illustration of the here described tissue formation and tensioning process. Following initial cellular spreading and adhesion (1), cells incrementally deposit tensioned collagen fibrils (black arrows, 2) which leads to a gradual increase in the total force resulting in a macroscopic contraction (3). By this, the amount of macroscopic force exceeds the sum of single cell forces or contributions from nonfibrillar ECM networks. The collagen deposition rate thus determines the quantity of macroscopic contraction and tensioning of regenerating tissues, which is important to restore their function. Increased contraction, however, is associated with pathologies such as fibrosis and cancer.

Together our findings point to an incremental process in which cells contract and stabilize the environment by transferring tension to collagen fibrils. Such a “slip and ratchet” model for tissue contraction has been proposed and discussed for many years but the data presented here give a first in vitro evidence for the validity of this model (**Figure** **6**).^3^ Our findings suggest that collagen fibrils, beyond the current understanding of wound contraction, play an important role in re‐establishing tension during tissue healing. The proposed amplification of cell forces that results from the storage of tensional force in extracellular collagen fibrils is expected to generate tissue tension significantly higher than the sum of cell traction forces alone.

However, it has to be pointed out that our interpretations are based on a 3D in vitro model system that comprises a number of limitations. Although we have demonstrated that the scaffold deforms elastically and maintains its properties throughout culture, we disregard microscopic variations (e.g., in wall thickness and pore diameter) that might affect cellular contractility and tensioning. This might be the case especially at early stages where a collective mechanosensation through long range fiber alignment is still missing. Furthermore, we do not take into account that cells and cell‐secreted ECM might represent elements carrying compressive loads during tissue contraction that are not considered in the analysis. Such factors are difficult to assess but might influence the intrinsic tensional state of the tissue beyond the scope of this study. The actual magnitude of cellular force multiplication through collagen fibrils might thus differ significantly from our estimations.

During tissue regeneration, fibroblasts undergo a temporary and potentially reversible transition into myo‐fibroblasts.^25^, ^39^ This is due to the presence of soluble factors such as TGF‐β driving epithelial to mesenchymal transition (EMT), collagen I/III production and α‐smooth muscle actin expression or an altered mechanical environment.^17^, ^40^, ^41^, ^42^ Myo‐fibroblast activation was described as a function of tissue stiffness and the occurrence of myo‐fibroblasts in vivo coincides with tissue stiffening and granulation tissue maturation.^4^, ^39^ We cannot exclude that fibroblasts were activated during scaffold‐based in vitro culture, e.g., in response to the time‐dependent increase of tissue stiffness. Donor‐dependent fibroblast activation might thus contribute to the large inter‐donor differences observed in macroscopic scaffold contraction (Figure 3c). As a further limitation of this study, such time‐dependent alterations of cell traction forces in their physiological environment were disregarded. The quantification of traction forces via 2D TFM performed here provides only limited information about the forces that cells exhibit in their physiological environment.^23^ However, the only twofold increase in single cell forces upon fibroblasts activation by TGF‐β compared to the large variation of scaffold contraction (up to 17‐fold) and of the amplification factor (up to 100‐fold between D1 with *A*_F D1, median_ = 0.23 and D3 with *A*_F D3,median_ = 23) indicate that cell activation alone cannot explain the observed differences in the scaffold contraction behavior. Even under consideration of the above‐mentioned limitations, the finding that scaffold contraction correlated linearly with collagen fiber density but not with single cell force strongly points toward collagen fibrils as an essential regulator and potential storage of cell traction forces during scaffold/tissue contraction. Myo‐fibroblast activation in vivo might further potentiate this effect since it not only increases cellular forces but also collagen secretion.^17^, ^41^

From the proposed model, a direct connection results between the directionality of tissue tension and collagen fibril alignment. This suggests that regenerative approaches should aim at controlling collagen fibril deposition not only for structural optimization of the extracellular matrix but also to restore, or prevent, specific states of tissue pretension, e.g., in musculoskeletal tissues. First examples how patterned biomaterials can be used to avoid uncontrolled collagen formation and scarring to support tissue regeneration are emerging.^30^

Apart from the suggested relevance of collagen fibrils for wound contraction, aberrant collagen deposition and alignment is also a hallmark of tissue fibrosis and cancer.^43^, ^44^, ^45^, ^46^, ^47^, ^48^, ^49^, ^50^ Based on the finding that contraction and collagen fibril deposition are mechanically linked rather than successive processes, we speculate that an unbalanced collagen fibril‐mediated tissue contraction might contribute to the development of pathological situations. Consequently, aberrant collagen deposition during prefibrotic or premalignant stages might contribute in the path toward scar or tumor manifestation. Such an assumption is supported by in vitro, but also human mammogram studies, which could relate a collagen‐rich tissue to an increased risk of developing breast cancer.^51^, ^52^ Although especially in malignant situations other factors (e.g., oncogene mutation, inflammatory response) are of highest relevance, altered collagen deposition rates would resemble a key link in transferring cellular alterations into a mechanical and structural constituent.

## 3 Conclusion

Together, we provide in vitro evidence that the storage of cell‐traction forces inside the cell‐secreted collagen fibril network represents a relevant mechanical component in tissue tensioning and contraction. A closer look at collagen fibrils as a key component in tissue tensioning might in future allow better control over pathological tissue alterations and motivate innovative approaches for successful tissue regeneration.

## 4 Experimental Section

*Cell Isolation and Culture*: Primary hdFs were isolated from human skin biopsy samples. Isolation of primary fibroblasts from patient‐derived material was approved by the Institutional Review Board of the Charité Berlin. All patients gave their written consent. Isolated cells were expanded in Dulbecco's Modified Eagle Medium (DMEM, # 11960‐044; Thermo Fischer) supplemented with fetal bovine serum (FBS, 10% (v/v), # S 0115; Biochrom AG), penicillin (100 U mL^−1^)and streptomycin (100 µg mL^−1^) (1% (v/v) # A 2213; Biochrom AG), and nonessential amino acids (NEA, 1% (v/v), # K 0293; Biochrom AG) at 37 °C with 5% CO_2_ in a humidified incubator. The hdFs were passaged when reaching confluence using 1 × Trypsin/EDTA (# 59418C; Sigma‐Aldrich).

*Scaffold Seeding and ECM Formation*: All experiments were conducted using hdFs between passages 4 and 9. Optimaix macro‐porous, porcine collagen I/III scaffolds were purchased from Matricel GmbH, Herzogenrath, Germany. Scaffolds were manufactured by a directional freezing and freeze‐drying process with additional chemical cross‐linking and used as a cell carrier.^28^ Three scaffold types with solid collagen contents of 1.1, 1.5, or 3.0% (w/w) in the initial aqueous suspension were used in this study. Cylindrical scaffolds (Ø 5 mm, 3 mm height) were prepared from sheets using a biopsy punch. Scaffolds were seeded by dip‐in‐uptake from a concentrated cell suspension (7500 cells µL^−1^). The samples were incubated for 1 h at 37 °C in a humidified incubator w/o additional medium to allow cell adhesion, successively rinsed in expansion medium to remove unattached cells and placed into a well‐plate overnight. The next day, the samples were washed once and further incubated with DMEM tissue growth medium supplemented with FBS (2% (v/v)), P/S (1% (v/v)), NEA (1% (v/v)), and l‐ascorbic acid (1.36 × 10^−3^ m) The medium was exchanged after 3, 7, and 10 d of culture.

*Decellularization*: Decellularization was performed in a Triton‐X100 (1% (v/v), #93443; Sigma‐Aldrich)/SDS (0.1% (w/v), #15553027; Thermo Fischer) detergent solution at room temperature (RT) under continuous agitation for 48 h and with 1 solution exchange after 24 h. The samples were washed in PBS and incubated with DNase I (700 U, # 10104159001; Roche) dissolved in PBS containing Calcium and Magnesium (# 14.040‐091, Thermo Fischer) for further 24 h at RT. Finally, samples were washed in PBS w/o Calcium and Magnesium (# 14.190‐094, Thermo Fischer).

*DNA Quantification*: DNA content was quantified using the CyQUANT Cell Proliferation Assay Kit (#C7026; Thermo Fischer) with an adopted protocol. In brief, samples were snap‐frozen on dry ice and shattered in the cold under liquid nitrogen conditions using custom‐made steel sticks and silicone pots and 1× Cell lysis buffer was added in the cold. The lysate was transferred to a test tube and centrifuged to pellet the scaffold debris. The supernatant was mixed in a 1:1 ratio with 2× GR working solution and the fluorescence was measured in a 96‐well plate at 485/530 excitation/emission using a plate reader (Infinite 200pro, Tecan). For calculation of DNA amount/sample, a DNA standard curve was created according to the manufacturer's instructions.

*SEM*: Scanning electron microscopy of freeze‐dried, gold‐sputtered samples was performed using the JCM‐600 (JEOL GmbH) device.

*Mechanical Testing*: Elastic moduli of collagen scaffolds were analyzed by mono‐axial compression testing of cubic samples using a BOSE ElectroForce Mechanical Test Instruments TestBench system equipped with a Model 31 Low load cell (Honeywell Corp.). Samples were compressed to 10% strain with a compression rate of 0.05 mm s^−1^ and the position was kept constant for 30 seconds at 0 and 10% strain position respectively. Before the measurement, samples were scanned to obtain the cross‐sectional area and height for the calculation of stress and strain values. Stress‐strain curves were calculated and plotted from measured load and the Young's Modulus *E* [Pa] was obtained from the linear region of the curve. Scaffold strain energy *U* for the different calculated for axial and radial scaffold orientation separately was calculated based on Equation (1)

(1) $$U=W=\frac{1}{2}\cdot F\cdot s$$

Hematoma was harvested during orthopedic surgery 6 ± 3 d postfracture and mechanically tested within 1 h after surgery. Collection of human fracture hematoma was approved by the Institutional Review Board of the Charité Berlin. All patients gave their written consent. Hematoma samples were compressed over 15% strain with a compression rate of 1 mm min^−1^. Young's Moduli were retrieved from the linear region of compression curves.

*Bioreactor System*: Permanent compressive loading with in situ monitoring of the mechanical properties was performed inside a custom‐made bioreactor system described previously.^33^ In brief, the system is composed of two separate compartments: a cell culture unit and a mechanical unit. The cell culture unit consists of a bioreactor chamber, a medium reservoir allowing gas exchange, and a micropump. It is combined with the mechanical unit allowing the application of defined loading patterns with online‐force measurements. To mimic the scaffold contraction during tissue growth, empty collagen scaffolds (Ø = 8 mm) were incubated in full fibroblast expansion medium and compressed in incremental steps of 45 µm d^−1^ up to 10 d reaching a total compressive strain of 13%. To assess the change of mechanical properties over time, compression tests were conducted after each increment (= 24 h). Therefore, the upper plunger returned to its zero position (= initial position) and samples were compressed three times over 15% strain with a compression rate of 0.05 mm s^−1^. The position was kept constant for 30 s at 0 and 15% strain position, respectively.

*Collagenase Degradation*: Decellularized samples were enzymatically treated with crude collagenase I (# 17100‐017, Thermo Fischer) as described previously.^53^ In brief, crude collagenase I was dissolved in Tris‐HCl (0.1 m, pH 7.4) supplemented with sodium azide (0.05 g L^−1^) and calcium chloride (5 × 10^−3^ m) and samples were treated with 1 U for 24 h at 37 °C.

*Western Blotting*: Whole tissue samples were frozen and mechanically minced using custom‐made steel pestle and silicone molds. Sample powder was mixed with a detergent buffer containing 4% CHAPS, 50 × 10^−3^ m TRIZMA base, 50 × 10^−3^ m KCl, and 20% wt/vol glycerol at pH 7.5, as well as proteinase and phosphatase inhibitor cocktail (Complete, PhosStop; Roche). Lysates were sonicated on ice and successively mixed with a solution containing 6.5 m urea, 2 m thiourea, and 5 × 10^−3^ m magnesium chloride in a 2 + 1 ratio. The supernatant was used for electrophoresis by mixing 4× sample loading buffer (Li‐Cor) and denaturation at 85 °C for 5 min. SDS‐polyacrylamide gel electrophoresis and western blotting was performed using the NuPAGE electrophoresis system (Thermo Fischer) according to the manufacturer's instructions. Proteins were transferred onto Protran nitrocellulose membrane (0.45 µm pore size, # GE10600003, Sigma‐Aldrich) and blocked after transfer using Odyssey TBS blocking buffer (#927‐50000, Li‐Cor). Primary antibodies for histone H3 (#4499, Cell Signaling), β‐actin (#8457, Cell Signaling) and collagen type I (#ab138492, abcam) were used according to the manufacturer's instructions. Secondary antibodies (antirabbit, #925‐32211, Li‐Cor) were incubated using 3% BSA/TBS‐T and signals were detected using the Odyssey infrared imaging system (Li Cor).

*Mass Spectrometry*: LC/ESI–MS was performed as described previously.^54^ In brief, decellularized scaffolds were washed using Ammonium bicarbonate (25 × 10^−3^ m; Sigma‐Aldrich)/Acetonitril Uvasol (2% (v/v); Merck) in water and twice with ammonium bicarbonate (25 × 10^−3^ m)/Acetonitril (50% (v/v)) in water at 37 °C. Afterward scaffolds were dehydrated with 100% acetonitrile at 37 °C. Samples were subjected to tryptic digestion at 37 °C over night (0.01 µg µL^−1^ Trypsin in 50 × 10^−3^ m ammonium bicarbonate; Promega). Peptides were extracted with trifluoridic acid (0.1% (w/v)) and directly analyzed by LC/ESI–MS. Peptides were separated (2–60% acetonitrile/ in 0.1% formic acid, flow rate 400 nL min^−1^) using an analytical UHPLC System (Dionex Ultimate 3000 RSLC, Thermo‐Fisher, Waltham, MA USA) and analyzed via ESI‐QTOF‐mass spectrometer (Impact II, bruker daltonics, Billerica, MA, USA). Mass spectra were evaluated using MASCOT software (version number 2.2, Matrix Science, Boston, MA, USA) automatically searching the SwissProt 51.9 database (553 474 sequences; 198 069 095 residues, Cambridgeshire, UK). MS/MS ion search was performed with the following set of parameters: i) taxonomy: homo sapiens (human) (20 172 sequences); ii) proteolytic enzyme: trypsin; iii) maximum of accepted missed cleavages: 2; iv) mass value: monoisotopic; v) peptide mass tolerance 10 ppm; vi) fragment mass tolerance: 0.05 Da; and vii) variable modifications: oxidation of Methionine. Only proteins with scores corresponding to *p* < 0.05 and with at least two independent peptides which were identified, were considered. Visualization of protein interaction networks was performed using String DB (http://string‐db.org) with high confidence interaction score (0.7) and experiments, databases, co‐expression and co‐occurrence as active interaction sources. GO enrichment analysis was performed using the open access platform Term Finder (http://go.princeton.edu/cgi‐bin/GOTermFinder) with cellular component as ontology aspect.

*Scaffold Contraction Analysis*: Scaffold contraction analysis was performed by scanning the samples at the respective time points in tissue growth medium using a digital scanner (1200 dpi, Epson perfection V200). The cross‐sectional area (*xy*) was calculated by manual contouring of the sample in the top view and the height by measuring the average distance between top and bottom of the sample in the side view. The scaffold contraction was calculated as the percentile difference in area (top) or height (side) over the time of tissue growth normalized to the initial area/height (Equation (2)).

(2) $$\varepsilon_{\mathrm{radial}}^{m}\left(\%\right)=\frac{\left(A_{0}-A_{m}\right)}{A_{0}}\times 100\;\;\varepsilon_{\mathrm{axial}}^{m}\left(\%\right)=\frac{\left(h_{0}-h_{m}\right)}{h_{0}}\times 100$$

*Immunofluorescent Staining and Imaging*: Samples were fixed in paraformaldehyde solution (4% (w/v); Sigma‐Aldrich) and the reaction was stopped with ammonium chloride (25 × 10^−3^ m (w/v)) buffered in PBS. Scaffolds were then incubated in gelatin (5% (w/v))/sucrose (5% (w/v)) buffered in PBS at 37 °C and successively transferred to 4 °C to allow solidification. The cylindrical samples were cut into halves and the gelatin was washed out with PBS at 37 °C. In order to obtain an even plane for imaging, the samples were finally processed by transferring them into cryomoulds, covering with Tissue‐Tek* O.C.T. Compound (Sakura Inc., #25608‐930) and snap‐freezing under liquid nitrogen conditions. The surface was prepared in a CryoStat (LEICA CM3050S). The following antibodies and probes were used for immuno‐fluorescent labelling: fibronectin (# ab23750; Abcam), α‐smooth muscle actin (#M0851; Dako), α‐rabbit IgG‐A88 (#A‐21206, Thermo Fischer), actin (Phalloidin‐Alexa633, # A22284, Thermo Fischer, Phalloidin‐Atto550, #19083, Sigma‐Aldrich), DNA (DAPI, # 62247, Thermo Fischer; Draq5 #424101, Biolegend). Microscopy was performed using a LEICA SP5 confocal microscope equipped with a Mai Tai HP multiphoton laser (Spectra Physics) and a 25‐fold water immersion objective. 620 µm × 620 µm × 52 µm volumes were recorded with a resolution of 0.6 µm × 0.6 µm × 4 µm voxel size. High‐resolution images for quantification of scaffold wall thickness were recorded using a 63‐fold water immersion objective with a resolution of 0.12 µm × 012 µm × 0.98 µm voxel size. Fibrillar collagen was detected by second harmonic generation with laser power and detection parameters kept constant for all measured samples (SHG, 910 nm excitation, 440–460 nm detection).

*Image Analysis*: All analysis steps of confocal microscopy images were performed in ImageJ. Collagen scaffold pore diameter was quantified by manual measuring using confocal planes of SHG tile scans. The thickness of collagen walls was quantified using BoneJ.^55^ For analysis of fibrillar collagen density, the scaffold pores were selected as a region of interest (ROI) and the sum of the signal intensity was normalized to the ROI volume. The orientation distribution of actin, fibronectin and newly formed fibrillar collagen inside the pores was quantified using OrientationJ.^56^ The anisotropy was quantified inside scaffold pores using FibrilTool.^57^ Cell numbers inside scaffolds were quantified based on cell density calculated from nuclei staining and successive particle counting using ImageJ. The amount of cells was converted into cell density and related to the total scaffold volume. Cell density heatmaps were generated by measuring cell density in a 200 µm grid pattern across whole sample cross‐sections.

*Histology*: Cryosections of human fracture hematoma were prepared using a CryoStat (LEICA CM3050S). Sections were air‐dried and stained with Sirius red solution (0.2% (w/v)) dissolved in saturated picric acid. Samples were further washed in acetic acid (0.5% (v/v)) followed by successive dehydration in 70–100% ethanol and xylol. Stained sections were imaged under a bright field microscope (Zeiss) at 5× magnification.

*Traction Force Microscopy*: Quantification of single cell forces and calculation of force fields and strain energy values from recorded image pairs was performed as described previously.^34^, ^35^ In brief: Clean coverslips (22 × 40 mm, Langenbrinck GmbH #01‐2240/1) were prepared by successive sonication in SDS (0.1% (w/v)), ddH_2_O, 70% ethanol and 100% for 30 min at 60 °C. Coverslips were activated using 3‐Aminopropyltrimethoxysilane (50% (v/v), APTMS, #281778; Sigma‐Aldrich) and glutaraldehyde (0.5% (v/v), #G5882; Sigma‐Aldrich). Polyacrylamide (PAA) gels were prepared on activated coverslips using FluoSpheres (0.1 µm, #F8800; Thermo Fischer) embedded into acrylamide (#161‐0140;Bio‐Rad), bis‐acrylamide (#161‐0142; Bio‐Rad). Polymerization was induced using ammonium persulfate solution (10% (w/v), # A3678; Sigma‐Aldrich) and tetramethylethylendiamin (TEMED, #161‐0800; Bio‐Rad). Polyacrylamide gels were rinsed with ddH_2_O and coated using 50 µL fibronectin solution (1 mg mL^−1^, #341635; EMD Milipore) in combination with UV‐light (10 mW cm^−2^ for 150 s) activated sulfosuccinimidyl 6‐(4′‐azido‐2′‐nitrophenylamino)hexanoate (Sulfo‐SANPAH, #22589; Thermo Fischer). The final elastic modulus of the PAA gels was validated by nanoindentation using the Piuma nanoindenter (Optics 11) with a 9 µm spherical cantilever tip. Coated substrates were washed with sterile PBS solution and seeded with hdFs at a density of 3000 cells cm^−2^. For TGF‐β or ascorbic acid prestimulation, cells were cultured 4 d prior to trypsinization either using 10 ng mL^−1^ TGF‐β1 (#100‐21, Peprotech) or 50 × 10^−6^ m ascorbic acid. The next day, cells were stained with cell tracker green (CTG, Thermo Fischer, # C7025) and cover slips were transferred into a custom made perfusion chamber and mounted on top of an inverted confocal microscope (Leica SP5) equipped with a 63× water immersion objective. Images were recorded with a spatial resolution of 240 nm, and a z‐resolution of 500 nm. Cells were removed by trypsinization and beads were recorded for marked cell positions to obtain image pairs. Calculations of strain energies per cell was based on Image‐J plugins for Particle Image Velocimetry and Fourier Transform Traction Cytometry with regard of the cell outline based on CTG signals.^58^

*Data Presentation and Statistics*: All data are shown as mean value with standard deviation. Box plots are shown as box with 25% and 75% for lower and upper limits. The mean value is indicated as a square and the median as horizontal line. Diagonal crosses indicate outliers. Statistical analysis was performed using the OriginPro 2015G (OriginLab Corporation) software. Two‐sided Mann–Whitney‐U statistical test was used for assessing significance levels with a Bonferroni correction for comparison of multiple groups. A value of *p* < 0.05 was considered as statistically significant. Different significant levels are indicated as: # *p* < 0.1, * *p* < 0.05, ** *p* < 0.01, *** *p* < 0.001.

## Conflict of Interest

The authors declare no conflict of interest.

## Supporting information

Supplementary
