# Supplementary material for: Collagen Fibrils Mechanically Contribute to Tissue Contraction in an In Vitro Wound Healing Scenario
Source: Adv Sci (Weinh). 2019 Mar 14;6(9):1801780. doi: 10.1002/advs.201801780 (PMC6498124; doi:10.1002/advs.201801780)
Supplement: Supplementary file 1 — Supplementary [file ADVS-6-1801780-s001.pdf]

Copyright WILEY-VCH Verlag GmbH & Co. KGaA, 69469 Weinheim, Germany, 2019.

ADVANCED SCIENCE
Open Access

Supporting Information

for *Adv. Sci.,* DOI: 10.1002/advs.201801780

Collagen Fibrils Mechanically Contribute to Tissue Contraction in an In Vitro Wound Healing Scenario

*Erik Brauer, Evi Lippens, Oliver Klein, Grit Nebrich, Sophie Schreivogel, Gabriela Korus, Georg N. Duda, and Ansgar Petersen**

Supporting Information

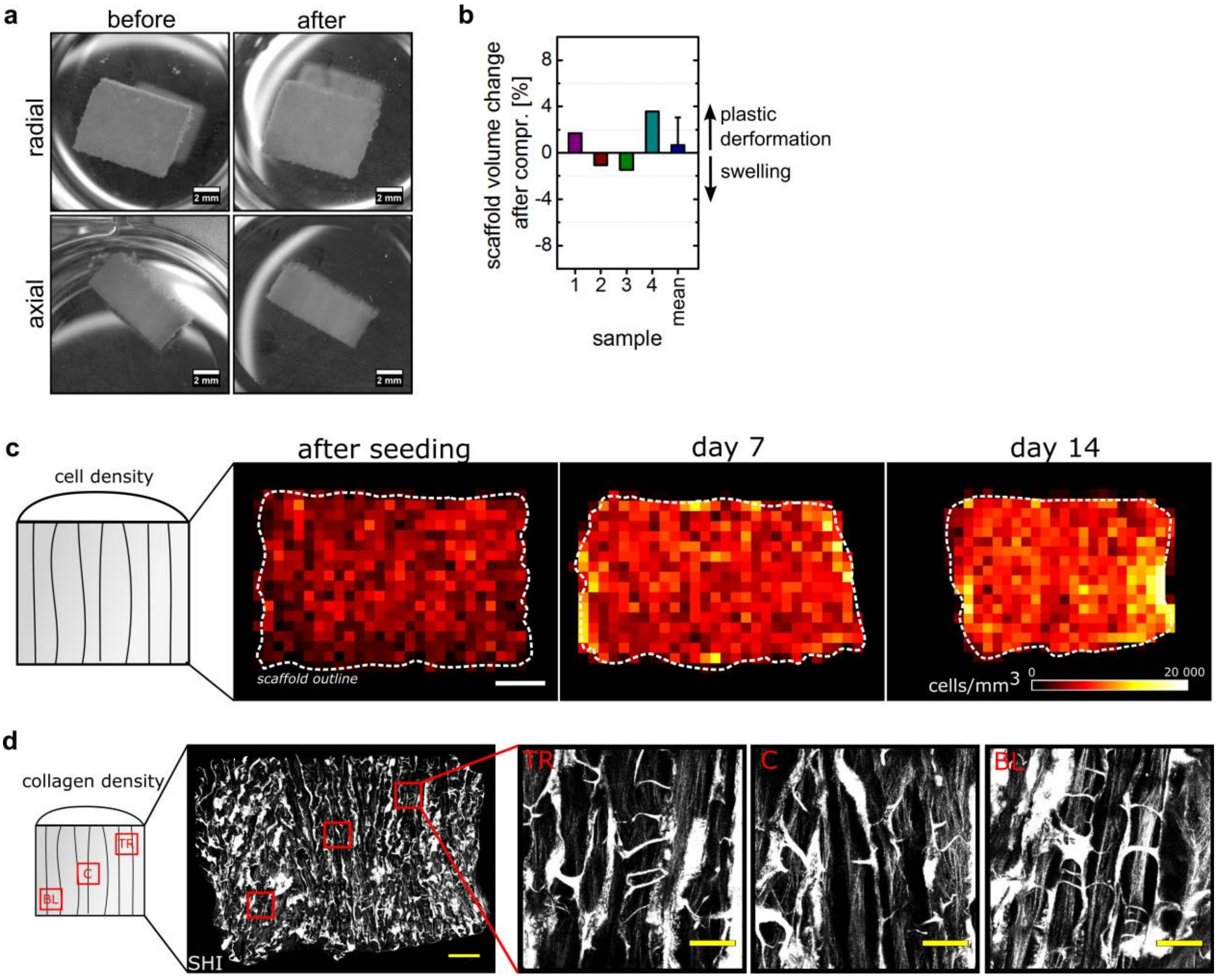

Figure S1: Maintenance of scaffold shape during repetitive compression & homogeneity of cell and ECM distribution. (a) Collagen scaffolds imaged in axial and radial direction before and after 150 cycles of repetitive compression **(b)** Calculation of volume change (initial volume – volume after compression testing) where a positive value indicates plastic deformation and a negative value scaffold swelling. **(c)** Heatmaps illustrating local cell density over the cross-section of scaffolds after seeding (day 0) and 7 or 14 days of culture. White dashed lines indicate the scaffold outline. Scale bar 1mm. Images were derived from representative samples. **(d)** SHI overview images with top-right (TR), center (C) and bottom-left (BL) close-up ROIs illustrating homogenous fibrillar collagen deposition throughout the sample. Scale bar 500µm (left) or 100µm (close-up zoom images). Images were derived from representative samples.

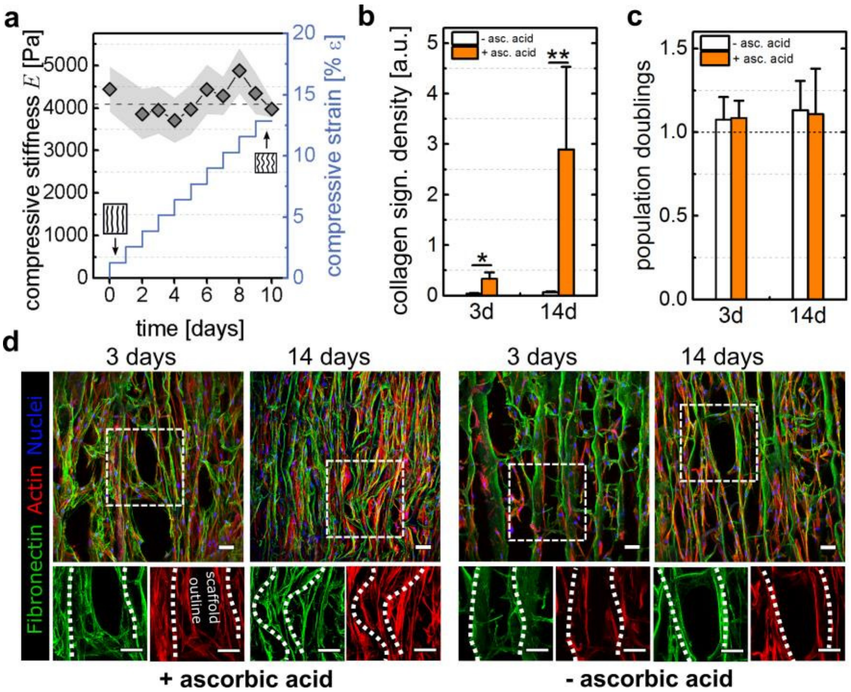

Figure S2: Depletion of ascorbic acid does not alter cell and soft ECM organization (a) In situ measurement of mechanical properties of empty scaffolds inside a bioreactor system undergoing permanent deformation over a time frame of 10 days. The blue axis indicates the incremental increase in compressive strain. n=2 **(b)** Quantification of SHG signal from samples cultured either in the presence or absence of ascorbic acid for 3 or 14 days (Mean ±S.D., n=4-9). **(c)** Population doublings of hdFs inside scaffolds over culture of 3 or 14 days based on an estimated original cell density of 7500cells/µl and the scaffold size at day 0 (Mean ±S.D., n=3-7). **(d)** Confocal images of samples cultured either in the presence or absence of ascorbic acid for 3 or 14 days. Samples were stained for fibronectin (green), actin (red) and cell nuclei (blue). White dashed lines in close-up zooms indicate scaffold outline. Scale bar 50µm. Significance levels via Mann-Whitney U test (two-sided) with Bonferroni correction for comparison of multiple groups. Significance levels indicate * $p<0.05$ and ** $p<0.01$.

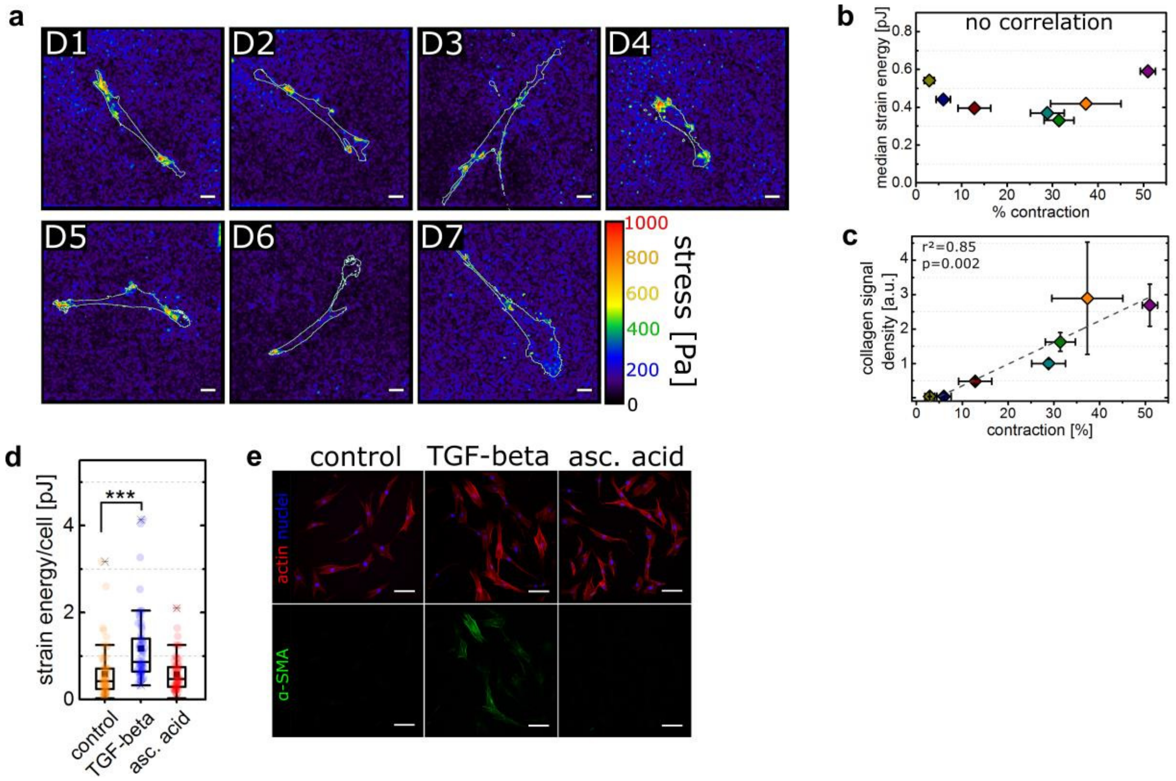

Figure S3: **(a)** Traction force magnitude maps of cells for the analyzed donors (D1-D7) including the cell shape as white outline. Scale bar 20µm. **(b)** 2D strain energy of single cells plotted against 3D scaffold contraction indicates no correlation. **(c)** collagen fibril density plotted against 3D scaffold contraction indicates a linear correlation after 14 days of culture**.** **(d)** Single cell strain energies measured by Traction Force Microscopy of at least 60 cells. Cells were pre-stimulated either with 10ng/ml TGF-β1 or 50µM ascorbic acid 4 days prior to TFM measurement. **(e)** Immunofluorescent staining of fibroblasts for alpha-smooth muscle actin (green) after days of stimulation either with 10ng/ml TGF-β1 or 50µM ascorbic acid. Cells were additionally stained for F-actin (Phalloidin, red) and nuclei (blue). Significance levels via Mann-Whitney U test (two-sided) with Bonferroni correction for comparison of multiple groups. Significance levels indicate ***$p<0.001$.

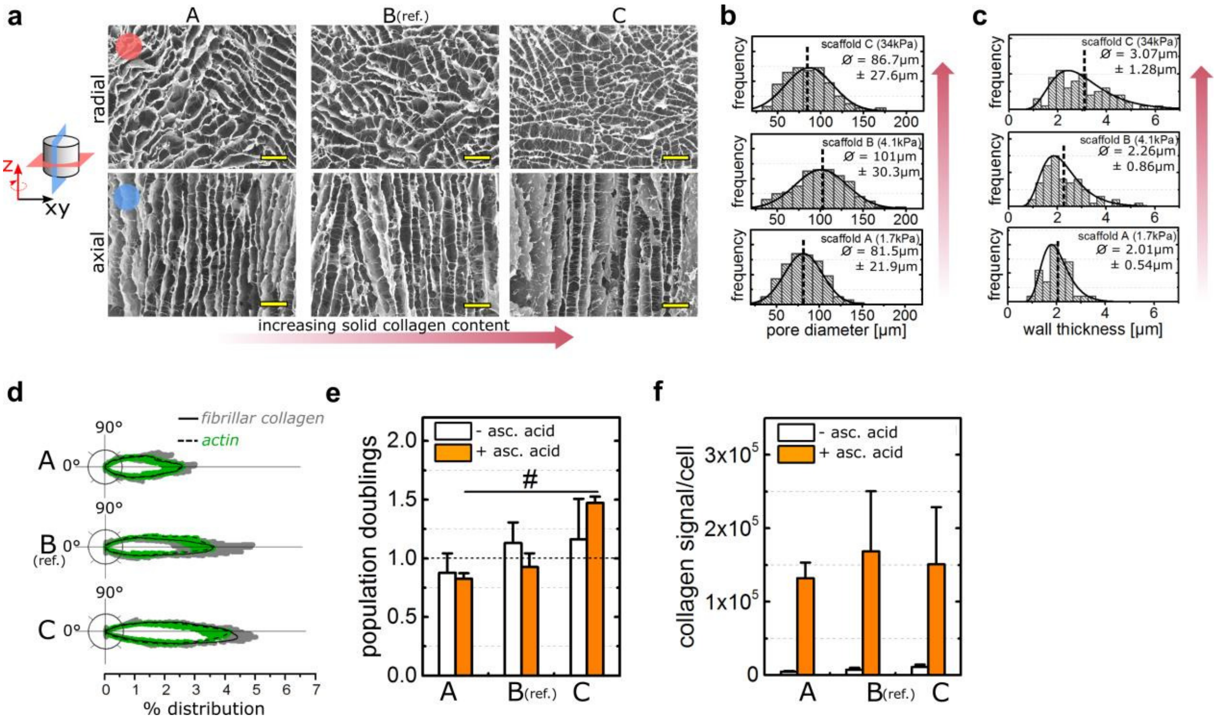

Figure S4: Scaffolds of varying collagen content do not differ in architecture. (a) SEM images of scaffold A, B and C recorded both in axial (blue) and radial (red) direction. Scale bar 250µm. **(b)** Histograms showing the distribution of pore spanning distance for scaffolds A, B and C. Fitted normal distribution curve is illustrated as black line. Black dashed line indicates the mean value. **(c)** Histograms showing the distribution of wall thickness for scaffolds A, B and C. Fitted normal distribution curve is illustrated as black line. Black dashed line indicates the mean value. **(d)** Circular plots of cellular actin signal and/or fibrillar collagen signal relative to a normal distribution after 14 days of culture inside scaffold prototypes A, B and C. Dark integrated or dashed lines indicate the mean with green (actin) or grey (fibrillar collagen) belt reflecting the standard deviation (Mean ±S.D., n=3-4). **(e)** Calculated population doublings of hdFs after 14 days of culture inside scaffold prototypes. The values are based on estimated initial seeding density and volume compared to quantified cell density and volume after 14 days of culture (Mean ±S.D., n=3-7). **(f)** Calculated fibrillar collagen signal per cell (n=3-7). Statistics are performed using the Mann-Whitney U test (two-sided). Significance levels are indicated as: # p<0.1.

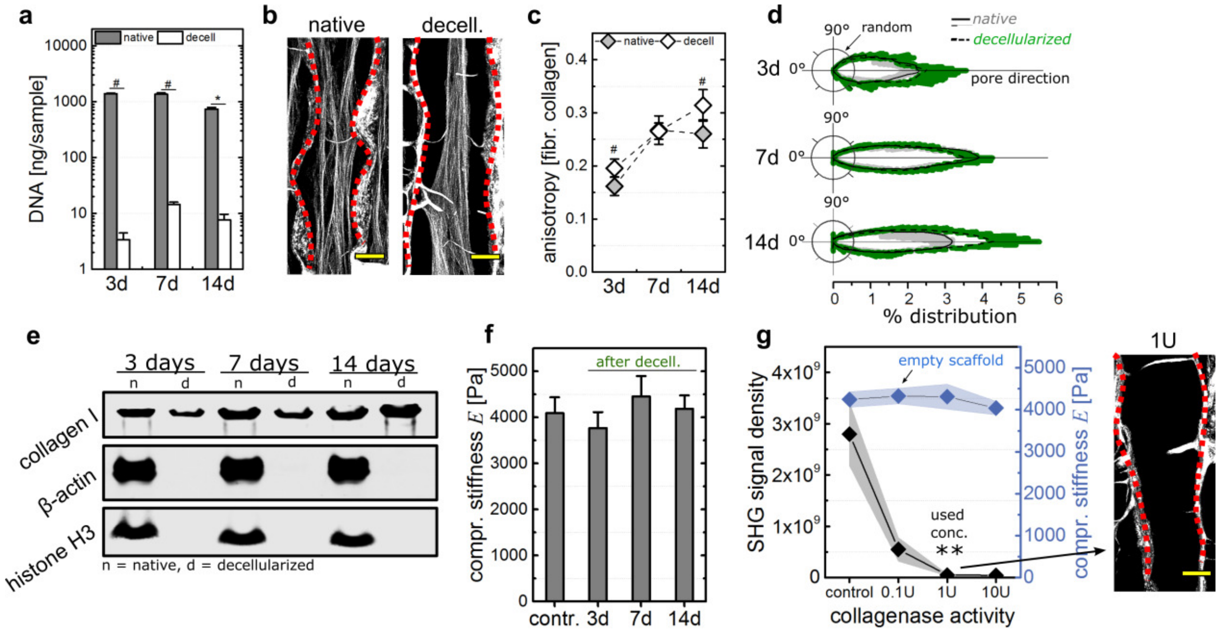

Figure S5: Decellularization does not affect fibrillar collagen (a) Quantification of DNA content before and after decellularization after 3,7 and 14 days of culture (Mean ±S.D., n=3-4). **(b)** SHG confocal images showing fibrillar collagen inside scaffold pores after 14 days of culture before (native) and after (decell.) decellularization. Red dashed lines indicate the scaffold wall outline. Scale bar 50µm. **(c)** Fibrillar collagen matrix fiber anisotropy before and after decellularization after 3, 7 and 14 days of culture (Mean ±S.D., n=3-4). **(d)** Circular plots of fibrillar collagen signal before (native, green) and after (decellularized, grey) decellularization after 3,7 and 14 days of culture. Integrated or dashed lines indicate the mean with grey or green belt as standard deviation. **(e)** Western blot of either native or decellularized whole microtissue samples after 3, 7 and 14 days of culture. Signals for collagen I, β-actin and histone H3 were detected illustrating the efficient removal of all cellular components but not collagen I. **(f)** Mono-axial compression testing of microtissues after decellularization after 3, 7 and 14 days of culture in comparison to empty scaffolds. **(g)** Effect of collagenase treatment for 24h at 37°C either on empty scaffolds for mechanical compression testing (blue) or decellularized samples after 14 days (black) at different concentrations. Significance for 1U collagen fibril density was tested against control samples. Right: Right: SHG image of 1 unit collagenase-treated decellularized samples after 14 days of culture. Scale bar 50µm. Statistics are performed using the Mann-Whitney U test (two-sided). Significance levels are indicated as: # p<0.1, * p<0.05, ** p<0.01

Table S1: Proteins identified by MS. Abbreviations: Score, Mascot protein score; emPAI, exponentially modified protein abundance index; OS, organism name; GN, gene name; PE, protein existence; SV, sequence version

sample	Score			emPAI			Description
	1	2	3	1	2	3	
	943	1244	1331	1.264	1.371	1.543	Collagen alpha-3(VI) chain OS=Homo sapiens GN=COL6A3 PE=1 SV=5
	497	489	930	0.505	0.468	0.595	Collagen alpha-1(VI) chain OS=Homo sapiens GN=COL6A1 PE=1 SV=3
	323	286		0.204	0.173		Collagen alpha-2(I) chain OS=Homo sapiens GN=COL1A2 PE=1 SV=7
	108	203	405	0.077	0.212	0.365	Collagen alpha-2(VI) chain OS=Homo sapiens GN=COL6A2 PE=1 SV=4
	185	199	75	0.160	0.136	0.064	Collagen alpha-1(I) chain OS=Homo sapiens GN=COL1A1 PE=1 SV=5
	44	149	67	0.038	0.101	0.064	Collagen alpha-1(XII) chain OS=Homo sapiens GN=COL12A1 PE=1 SV=2
	137	84	32	0.160	0.066	0.032	Thrombospondin-1 OS=Homo sapiens GN=THBS1 PE=1 SV=2
	87	74		0.077	0.032		Actin, aortic smooth muscle OS=Homo sapiens GN=ACTA2 PE=1 SV=1
		62			0.032		Histone H2A type 1-A OS=Homo sapiens GN=HIST1H2AA PE=1 SV=3
	96	59	55	0.077	0.032	0.032	Histone H4 OS=Homo sapiens GN=HIST1H4A PE=1 SV=2
		54			0.032		Decorin OS=Homo sapiens GN=DCN PE=1 SV=1
		53	61	0.000	0.066	0.032	Histone H3.3C OS=Homo sapiens GN=H3F3C PE=1 SV=3
	75	50	62	0.077	0.032	0.064	Fibrillin-1 OS=Homo sapiens GN=FBN1 PE=1 SV=3
		45	48		0.032	0.032	Myosin-9 OS=Homo sapiens GN=MYH9 PE=1 SV=4
	51	39	304	0.038	0.032	0.133	Periostin OS=Homo sapiens GN=POSTN PE=1 SV=2
	33	35		0.038	0.032		Histone H2B type 1-A OS=Homo sapiens GN=HIST1H2BA PE=1 SV=3
	45	45		0.038	0.032		Collagen alpha-1(III) chain OS=Homo sapiens GN=COL3A1 PE=1 SV=4
		40			0.032		KN motif and ankyrin repeat domain-containing protein 2 OS=Homo sapiens GN=KANK2 PE=1 SV=1
	31	39		0.038	0.032		Elastin OS=Homo sapiens GN=ELN PE=1 SV=3
	131			0.077			Histone H2A type 1-B/E OS=Homo sapiens GN=HIST1H2AB PE=1 SV=2
	63			0.038			Mimecan OS=Homo sapiens GN=OGN PE=1 SV=1

Table S2: Gene ontology (GO) annotation (cellular component, p<0.01).

GO_ID	Term	Frequency (cluster)	Corrected p-value	Annotated genes
GO:0031012	extracellular matrix	71.4%	1.76e-17	COL1A2, MYH9, ELN, HIST1H4A, COL3A1, THBS1, POSTN, FBN1, COL6A2, COL12A1, COL1A1, OGN, COL6A1, COL6A3, DCN
GO:0005578	proteinaceous extracellular matrix	57.1%	3.43e-14	COL1A2, ELN, COL3A1, POSTN, COL6A2, FBN1, COL12A1, COL1A1, OGN, COL6A1, COL6A3, DCN
GO:0005581	collagen trimer	38.1%	2.24e-12	COL1A2, COL3A1, COL6A2, COL12A1, COL1A1, COL6A1, COL6A3, DCN
GO:0005788	endoplasmic reticulum lumen	42.9%	8.07e-10	COL1A2, COL3A1, THBS1, COL6A2, FBN1, COL12A1, COL1A1, COL6A1, COL6A3
GO:0044421	extracellular region part	85.7%	2.19e-08	COL1A2, ELN, HIST1H2AA, COL3A1, THBS1, COL12A1, HIST1H2AB, ACTA2, MYH9, HIST1H4A, COL6A2, FBN1, POSTN, COL1A1, OGN, COL6A1, COL6A3, DCN
GO:0005589	collagen type VI trimer	14.3%	9.15e-08	COL6A1, DCN, COL6A3
GO:0098647	collagen beaded filament	14.3%	9.15e-08	COL6A1, DCN, COL6A3
GO:0005615	extracellular space	81%	1.52e-07	COL1A2, HIST1H2AA, COL3A1, THBS1, COL12A1, HIST1H2AB, ACTA2, MYH9, HIST1H4A, COL6A2, FBN1, POSTN, COL1A1, OGN, COL6A1, COL6A3, DCN
GO:0044420	extracellular matrix component	28.6%	1.94e-07	FBN1, COL1A2, COL12A1, ELN, COL1A1, COL3A1
GO:0005576	extracellular region	85.7%	5.30e-07	COL1A2, ELN, HIST1H2AA, COL3A1, THBS1, COL12A1, HIST1H2AB, ACTA2, MYH9, HIST1H4A, COL6A2, FBN1, POSTN, COL1A1, OGN, COL6A1, COL6A3, DCN
GO:0000786	Nucleosome	23.8%	9.84e-07	HIST1H2BA, HIST1H4A, HIST1H2AA, HIST1H2AB, H3F3C
GO:0044815	DNA packaging complex	23.8%	1.57e-06	HIST1H2BA, HIST1H4A, HIST1H2AA, HIST1H2AB, H3F3C
GO:0005583	fibrillar collagen trimer	14.3%	2.00e-05	COL1A2, COL1A1, COL3A1
GO:0098643	banded collagen fibril	14.3%	2.00e-05	COL1A2, COL1A1, COL3A1
GO:0070062	extracellular exosome	61.9%	4.98e-05	ACTA2, COL1A2, MYH9, HIST1H4A, HIST1H2AA, THBS1, FBN1, COL6A2, COL12A1, OGN, HIST1H2AB, COL6A1, COL6A3
GO:1903561	extracellular vesicle	61.9%	5.38e-05	ACTA2, COL1A2, MYH9, HIST1H4A, HIST1H2AA, THBS1, FBN1, COL6A2, COL12A1, OGN, HIST1H2AB, COL6A1, COL6A3
GO:0043230	extracellular organelle	61.9%	5.42e-05	ACTA2, COL1A2, MYH9, HIST1H4A, HIST1H2AA, THBS1, FBN1, COL6A2, COL12A1, OGN, HIST1H2AB, COL6A1, COL6A3
